# Research on How Emotional Expressions of Emotional Labor Workers and Perception of Customer Feedbacks Affect Turnover Intentions: Emphasis on Moderating Effects of Emotional Intelligence

**DOI:** 10.3389/fpsyg.2018.02526

**Published:** 2019-01-04

**Authors:** Young Hee Lee, Suk Hyung Bryan Lee, Jong Yong Chung

**Affiliations:** ^1^R&SD Strategy Center, Industry-Academy Cooperation Foundation, Chungwoon University, Incheon, South Korea; ^2^School of Integrated Technology and Entrepreneurship, Chungwoon University, Hongseong, South Korea

**Keywords:** emotional labor, emotional expression, perception of customer feedback, emotional exhaustion, emotional intelligence

## Abstract

Previous studies have used various external variables and parameters as well as moderator variables such as emotional intelligence have been to understand emotional labor and its related problems. However, a comprehensive model to study such variables’ correlations with each other and their overall effect on emotional labor has not yet been established. This study used a structural equation model to understand the relationship between employees’ expression of emotional labor and perception of customer feedbacks. The study also looked at how the perception of customer feedback affects emotional exhaustion in order to understand how emotional exhaustion affects job satisfaction and turnover intentions. Further, in order to fully understand the effects of emotion on emotional labor at the service contact points, this study developed and tested a model of emotional labor with four factors of emotional intelligence as moderating factors. Five hundred and seventy nine emotional labor workers in service industries in the United States were collected and 577 valid survey results have been analyzed. The result shows that there exists moderating effects of emotional intelligence on how employees’ Deep Acting and Surface Acting recognize customers’ reactions, both positive and negative, that would affect employees’ Emotional Exhaustion and Job Satisfaction, and hence, Turnover Intention. The result suggests that employees with better understanding of their own emotions, although they are more likely to recover from emotional exhaustion, experience a greater negative effect when there is a discrepancy between what they feel and how they should act.

## Introduction

The growth of the service industry has prompted the importance of employees’ emotional labor, and it has received increased attention from various fields including organizational behavior and organizational psychology (Grandey and Gabriel, 2015). A number of studies have found that emotional labor required by organizational norms can negatively affect individual well-being (Hülsheger and Schewe, 2011). Grandey (2000) stated that emotional labor can negatively affect workers’ job satisfaction, and the job satisfaction has strong implications for attendance, turnover, sabotage, job performance, and the mental and physical health of employees (Miao et al., 2017).

Emotional labor also indirectly affects employees by negatively affecting the performance of the organization. Among a range of factors leading to turnover intention, burnout has been found to play a significant role (Kilo and Hassmén, 2016), and surface acting contributes to the burnout, job dissatisfaction, and turnover intention (Lee and Chelladurai, 2018). Departures of skilled labor workers would cause additional expenses in recruiting, hiring, and training of new employees. It will also have negative effects on the service quality of the organization. Therefore, higher turnover rate would negatively affect the organizations business performances. In turn, it will negatively affect the job stability of the remaining employees. Therefore, in order to improve corporate performance, increase employee motivation and enhance employee’s job satisfaction, it is important to address employees’ emotional labor issues.

As the frontline employees are “barometers of the business” (Dagger et al., 2013, p. 498), especially in service industry, the organizations are trying to provide various supports for their employees at the service contact points (Cohen and Wills, 1985; Ashforth and Humphrey, 1993; Rhoades and Eisenberger, 2002; Kim et al., 2017). Social support is one of the supports that can be provided for the employees as it has long been identified as a resource that enables people to cope with stress (Fenlason and Beehr, 1994; Hobfoll, 2002). Social support is loosely defined as “the availability of helping relationships and the quality of those relationships” (Leavy, 1983, p. 5), and it could be provided by coworkers, supervisors, and the organization.

Lately, a number of studies put on more emphasis on the role of emotional reactions and interpersonal aspects (Mearns and Cain, 2003; Ullrich et al., 2012) as emotions are common among customers in service encounters (Gabbott et al., 2011; Strizhakova et al., 2012). Further, many studies have emphasized the importance of emotional intelligence which refers to employees’ ability to control their emotions and empathize with customers. Specifically, Mayer stated that “the ability to perceive emotions, integrate emotion to facilitate thinking, understand, and manage emotions to promote personal growth are paramount to professional achievements and mental health and well-being” (Mayer et al., 2016).

As an individual’s success at work is 80 percent dependent on emotional quotient and only 20 percent dependent on intelligence quotient (Goleman, 1998), it is important to address how Emotional intelligence affects the emotional labor. Emotional intelligence is found to be negatively associated with burnout, and it could therefore be a potential protective factor (Chan, 2006; Mikolajczak et al., 2007). Zysberg et al. (2017) found the similar result and stated that emotional intelligence can be inversely associated with burnout, a condition brought about by lack of effective emotional regulation and management. Emotional exhaustion is affected by employees’ emotional expression in the service encounter, and emotional intelligence of the individual will play a role in controlling these effects (Morris and Feldman, 1997; Park, 2018). As emotional intelligence is known to be improved by learning, experience, and training (Gardner, 1995), finding ways to enhance employees’ ability to control their emotions and empathize will help them.

Due to the importance of emotional intelligence as a personal resource to facilitate processing emotions into effective behavior patterns, Laborde et al. (2016) suggested that the organizations “should implement emotional intelligence training workshops for coaches to help them engage in emotional labor that benefits both themselves and the organizations.” However, although there have been various researches that looked into the effects of emotional intelligence on emotional labor, a comprehensive model to study the effects of the sub-dimensions of emotional intelligence has not yet been established.

Wong and Law (2002) extended the concept of emotional intelligence defined by Salovey and Mayer (1990), and refined the framework to categorize the sub-dimensions of emotional intelligence as perceiving, understanding, managing, and using emotions. Each of these sub-dimensions has different characteristics from each other. Further, due to their different characteristics, how each dimension can be strengthened and enhanced would be different from each other as well. Therefore, in order to understand how emotional intelligence affects emotional labor and suggest how emotional labor could be properly managed, how each sub-dimension of emotional intelligence affects emotional labor should be investigated instead of looking at the overall effect of emotional intelligence on emotional labor.

This study aims to suggest how employees’ turnover intention could be lowered in order to enhance organizational performance as well as their individual happiness. Therefore, this study adapted two main perspectives. The first is to look for solutions through the governing principles that control workers’ emotional expressions. The second is to look for solutions through individually controlled emotional intelligence. In order to accomplish these goals, this study used a structural equation model (SEM) to test the structural relationship among Emotional Exhaustion, Job Satisfaction, and Turnover Intention with Verbal and Non-Verbal Expressions, Customer Feedbacks, Surface Acting, and Deep Acting as observed variables. Further, the SEM was applied to the framework of Emotional Labor Model developed by Grandey and Gabriel (2015) to test the moderating effects of sub-dimensions of emotional intelligence on each stage from Emotional Labor through Emotional Exhaustion and Job Satisfaction to Turnover Intention. Identifying the moderating effects of each sub-dimension of emotional intelligence at each stage would be helpful in suggesting the solutions to employees’ emotional problems which would, in turn, reduce their turnover intention.

The results of this study would suggest that effective management of emotions can increase the satisfaction of the customers on the services, reduce emotional exhaustion of employees, and enhance employees’ job satisfaction, thereby lowering their turnover intention.

## Theoretical Background and Hypothesis Development

### Service Encounter

A service encounter occurs every time a customer interacts with the service organization. Specifically, the term ‘service encounter’ is used to indicate face-to-face interactions between a buyer and a seller in a service setting. These interpersonal exchanges can strongly influence customer satisfaction (Solomon et al., 1985).

Shostack (1985) added that service encounter is not only the interaction between the service provider and the customer, but also the period of interaction. In other words, the concept of service encounter is defined comprehensively by adding factors such as material, convenience, time, and space to human interaction. On the other hand, Keaveney (1995) referred to service encounter as the human interaction between the customer and the service employee, emphasizing that the human factor of the employee and the customer is the most important at that point.

The customer takes a variety of emotions and attitudes through communication with the employee while the service is being provided, and the employees and customers are highly interdependent in the service encounter (McCallum and Harrison, 1985).

### Emotional Labor

Emotional labor is generally defined as the act of expressing organizationally desired emotion during the service transactions, and is caused by the difference between the actual emotional state experienced by the employee and the emotional expression required by the organization’s emotional expression norms for effective job performance (Hochschild, 1983). Emotional labor is similar and consistently presented regardless of the differences of various cultures (Grandey et al., 2010).

Emotional labor is a type of labor in which emotional management activities for producing and maintaining a specific emotional state required by the job account for more than 40% of the jobs (Hochschild, 1979), and Emotional labor workers express certain emotions in the workplace, regardless of actual emotional experiences, in order to successfully follow the job demands (Hochschild, 1983). Jobs that require emotional labor include call center representatives, department store sales, flight crew, nurses, etc. (Hochschild, 1983; Brotheridge and Grandey, 2002; Williams, 2003).

Morris and Feldman (1996) defined emotional labor as effort, planning and control needed to express emotions demanded by firms while emotional workers are serving customers, and Grandey (2000) defined emotional labor as an effort to comply with organizational or vocational expression rules. Emotional labor is generated by controlling emotional expression during work (Lam and Chen, 2012), and it has been shown to affect counterproductive work behavior (Raman et al., 2016), service quality and customer satisfaction. Emotional labor workers conduct their duties through face-to-face conversations, voices, and actively expressed emotions in order to influence other people’s emotions, attitudes, and behaviors.

The effects of emotional labor within the organization are largely negative. These adverse effects directly affect the employees in the service encounters and spread to customers and organizations. Employees experience stress, depression, panic disorder, psychological distress, and job dissatisfaction. For the organizations, negative effects are exacerbated by employee dissatisfaction, performance reduction, and turnover. Further, emotional labor causes service quality deterioration, which leads to negative consequences such as decreased satisfaction with service and increased complaints by customers. Therefore, the emotions of the employees who represent the company in the closest position to the customers should be managed as an important variable in order to ensure the effectiveness of the organization.

Therefore, better understanding of emotional labor would provide meaningful insights to maintain customer’s favorable attitude, improve the service quality of employees, and lower the turnover intention by managing appropriate emotional expressions at the service contact points.

### Employees’ Emotional Expressions

#### Surface Acting and Deep Acting

Hochschild (1979, 1983) stemmed her dimensions of the emotional labor based on the approach of performing, and depicted two kinds of emotional labor. The first is Surface Acting. It refers to worker’s recreating of feelings, that are not really felt, by changing their outward appearance such as facial gesture, motions, or voice tone, when displaying required feelings. The second is Deep Acting. It happens when representative’s sentiments don’t fit the circumstance; then they utilize their preparation or past experience to express fitted feelings. Although Ashforth and Mael (1989) added another dimension, Genuine Emotion, to Hochschild’s work and suggested three main dimensions, Emotional labor has been mainly studied in two ways: Surface Acting and Deep Acting (Hochschild, 1983; Grandey, 2000; Brotheridge and Lee, 2003; Monaghan, 2006; Johnson and Spector, 2007).

Researchers found that Surface Acting has more negative effects on employees than Deep Acting. For example, employees smile and provide customers with services in kind voice even when they have negative feelings. Such a discrepancy in feelings often leads to emotional conflicts that will have a negative impact on employee job satisfaction. It also leads to emotional exhaustion and burnout for workers (Ashforth and Humphrey, 1993). On the other hand, Deep Acting refers to one’s effort to consciously modify feelings in order to express the desired emotion (Hochschild, 1983). It has a positive effect on the individual’s job satisfaction as well as the organization’s performance, accompanied by a positive result in interaction with the customer.

Both Surface Acting and Deep Acting are artificial rather than natural behaviors. However, while Surface Acting is a false emotional state that faithfully expresses the organization’s expression norm but does not actually control the inner emotional state, Deep Acting is the actual emotional state that attempts to match the organization’s expression norm with itself (Diefendorff et al., 2005). As a result, while employees in Deep Acting have a high level of job satisfaction and organizational satisfaction, Surface Acting negatively affects job performance because it is based on surface norms without controlling the inner feelings of employees. For example, Larson and Yao (2005) find that Deep Acting facilitates communication and more genuine forms of empathy toward patients, leading to higher levels of professional satisfaction.

Deep Acting may serve as a buffer against negative moods (Judge et al., 2009; Polletta and Tufail, 2016), and it has been found to reduce emotional exhaustion and emotional dissonance (Brotheridge and Lee, 2003; Kim and Kim, 2018). However, the employees in Deep Acting have negatively affected personal accomplishment, and it leads to conflicting results with surface behavior (Kruml and Geddes, 2000; Brotheridge and Grandey, 2002; Johnson and Spector, 2007). These results suggest that the consequences of emotional labor may depend on the type of emotion strategy utilized as well as the context from which it is performed (Grandey and Gabriel, 2015).

#### Verbal Expression and Non-verbal Expression

Communication is an important means of interactions that controls and links one’s relationship with the others as a member of the society (Gabbott and Hogg, 2001). In service encounters, Non-Verbal Expression and Verbal Expression both are major factors affecting customer satisfaction and job performance (Gayathridevi, 2013). As communication between employees and customers in service encounters could be a differentiating factor that separates a company from its competitors, it is important to manage the verbal and non-verbal communication performed by employees in order to improve the quality of interaction between employees and customers.

When customers choose products and services, they communicate with the company’s front line employees instead of the company itself. Therefore, the emotional response of the customer depends on the communication methods used by the employees at the service contact point where many interactions between the customer and the employee occur (Park et al., 2012). However, verbal and non-verbal efforts of employees to communicate with customers are, by nature, emotional labor from the employees’ standpoint. Thus, it is important to understand the interaction between the customer and employee at the service contact point.

From customers’ point of view, employees’ Verbal and Non-Verbal Expressions are considered as their Emotional Expression. Emotional Labor model developed by Grandey and Gabriel (2015) uses both Emotion Regulation (Surface Acting and Deep Acting) and Emotion Performance (Verbal and Non-Verbal Expression). This study would use such a framework to fully understand Emotional Labor and how its variables are structurally related.

##### Verbal expression

The linguistic communication is an important factor that can never be overlooked in the service environment, and in the relationship between the service provider and the customer. When delivering products and services, employees communicate information about products and services to customers through language. Language is the center of communication in human relationships and is the transmission and expression of meaning (Sommerville, 1982).

Linguistic communication is one of the systems promised by our society that individuals use to express their thoughts and feelings. Therefore, clear and rapid information transfer is an important clue to customer satisfaction (Kim, 2005), and there is a tendency for intimacy and trust to be increased through the communication process (Kim, 2007). In other words, intimacy and comprehension between employees and customers are increased by sending and receiving the necessary meanings between each other (Jandt, 1998). Employees can only reduce the barriers that may arise in the communication process by fully taking the customer’s situation into account (Bugental et al., 1970; Dodd, 1998).

Through linguistic communication, employees clearly communicate their desire for customers, and they understand what they need to do and respond to them, thereby creating mutual trust and intimacy, and eventually lead to a positive response from customers (Kim, 2005). Reliable verbal communication can maximize message delivery (Jones and LeBaron, 2002). Furthermore, Berko et al. (2016) noted that linguistic communication is important in corporate marketing activities and that it is directly linked to corporate trust formation. Marketing is closely related to the delivery of product descriptions and services, and marketing activities are mostly conducted in language.

##### Non-verbal expression

Non-verbal communication is a means of expressing oneself using space, time, and body movements (Dodd, 1998). Goldhaber (1982) defined non-verbal communication as all the silent messages except the language. In other words, non-verbal communication is the interaction of senders and recipients in a communication context, with the exception of language (Samovar et al., 1981).

Verbal communication is necessarily accompanied by non-verbal elements and sometimes non-verbal communication plays a more decisive role than verbal content. Birdwhistell (1955) argued that, in the communication process, 35% of linguistic messages and 65% of non-verbal messages are delivered. A person reads or expresses emotions or intention through non-verbal cues in situations where there is a limit to expressing them only by the use of language in a communication and a large amount of information is acquired in a short time (Stiff et al., 1994). Non-verbal communication also has the function of controlling social interaction. The beginning and end of conversations, the order of utterances, etc. can be controlled through non-verbal signals (Richmond et al., 1991).

Studies on communications have shown that non-verbal communication delivers more meaning faster than language and conveys feelings and emotions more accurately (Delmonte, 1991). Non-verbal communication narrows the psychological distance between employees and customers, increasing positive emotions such as pleasure and satisfaction, and helping to interact with linguistic communication (Sundaram and Webster, 2000).

Sundaram and Webster (2000) classified non-verbal communication into kinesics, proxemics, paralanguage, and physical appearances. Employees must maintain facial expressions with a bright smile (Burgoon et al., 1990), since the emotionless expression of the employees can cause discomfort to the customers. In other words, the face is the most important part that can contain a lot of information. It is called a multi-message system (Argyle, 1975) that can read information about personality, interest, reaction and emotion in facial expression. Looking elsewhere or a failure to pay attention to the customer may be conveyed to the customer in a feeling of indifference and neglect (Leigh and Summers, 2002).

On the other hand, the frequent convergence of employees and customers in service encounter increases the intimacy with each other (Wainwright, 1999). In addition, nodding or proper tilting of the customer’s words in the body language can be seen as an act of showing interest to the customer. This body language is one of the important forms of non-verbal communication that can convey interest, intimacy, benevolent feelings, and trust to customers (Mehrabian and Williams, 1969; Burgoon et al., 1990). Overall, individuals tend to rely on non-verbal communication in order to understand the emotion, while they rely on verbal communication in order to understand the thoughts (Hall and Schmid Mast, 2007).

### Customer Feedback (Positive/Negative)

We experience emotions, whether they are perceived or not, at every moment in various situations. In general, emotion is generally considered to be composed of two sub-factors that are correlated but independent: positive affect and negative affect. According to Watson et al. (1988), positive affect refers to emotions such as joy, pleasure, satisfaction, and happiness. Negative affect refers to feelings such as anger, fear, sadness, and guilt.

Emotions themselves refer to mental health (Diener, 1984). As static emotion promotes individual’s health and has the potential to bring about various physical, psychological and social changes, many scholars actively study the relationship between static emotional and physical health and psychological well-being (Lyubomirsky et al., 2005). In contrast, inadequacy has been widely used as a key indicator of mental health, and many studies have addressed the relationship between such negative emotions and problem behaviors such as psychopathology, suicide, assault, and stress (Sung and Gwon, 2010).

These emotions are also the result of actions or cognitive processes, and they are also the cause of behavior itself as well as cognition (Park and Lee, 2016). According to Pugh (2001), emotions of customers are behavioral responses caused by external environmental stimuli and they cause behavioral change according to the situation. The employees in service encounter can communicate their feelings to the customers during the interaction with the customers, and these feelings that are conveyed to the customers cause the positive or negative feelings of the customers.

In service encounter, it is important for employees to understand whether the customer’s response in the form of emotional expression is positive or negative. Because of the differences in experiences and values that customers and employees have, perceived meanings of emotional expressions might be different between customers and employees, which may lead to problems. The service provider could prevent such problems that may arise in communication process by conveying the message in full consideration of the customer’s situation (Dodd, 1998).

This study will focus on the Customer Feedback perceived by employees that occur in service encounter. Identifying how emotional labor of the employees is related to Customer Feedback would be helpful in suggesting effective ways to reduce emotional labor in service encounter. In particular, it is expected that more positive results from service encounter could be expected when Customer Feedback, whether it is positive or negative, are properly confirmed. So we propose the following hypothesis to be tested in this study.

Hypothesis 1: Employees’ emotional expressions will affect perception of customer feedback.
[H1_a1] Surface Acting → Customer Feedback (Positive)
[H1_a2] Deep Acting → Customer Feedback (Positive)
[H1_a3] Non-Verbal Expression → Customer Feedback (Positive)
[H1_a4] Verbal Expression → Customer Feedback (Positive)
[H1_b1] Surface Acting → Customer Feedback (Negative)
[H1_b2] Deep Acting → Customer Feedback (Negative)
[H1_b3] Non-Verbal Expression → Customer Feedback (Negative)
[H1_b4] Verbal Expression → Customer Feedback (Negative)

### Emotional Exhaustion

Emotional exhaustion refers to exhausted and depleted emotions due to work and is a chronic response to work stress situations that are associated with conceptually high levels of human contact (Ryan, 1971; Maslach, 1982; Mikolajczak et al., 2007; Zhao et al., 2018). Maslach (1982) described emotional exhaustion as a negative self-concept, a negative attitude toward work, and physical and emotional exhaustion symptoms including loss of interest or feelings to the customer. It is defined as the negative psychological experience that a person in charge of a job with high interpersonal contact experiences is exposed to a long time stressor (Maslach and Schaufeli, 1993).

Emotional exhaustion is caused by persistent and excessive emotional labor. Hochschild (1983) explained that emotional labor causes negative consequences such as emotional inactivation stress, physical exhaustion, emotional exhaustion, and absenteeism. Grandey (2000) also said that the emotional adjustment and social needs of emotional labor workers cause job exhaustion. In order to alleviate this emotional depletion, a phenomenon of de-personification that seeks psychological distance from others appears. When this de-personalization becomes severe, it leads to a decrease in the sense of self-fulfillment that a person feels no more efficient in performing tasks related to customers or performing their jobs (Maslach, 1982).

The surface behavior follows the norm of the emotional behavior manual given by the organization regardless of the employee’s own feelings (Hochschild, 1983). When such a surface behavior is performed and the different emotions than the workers’ true feelings should be expressed, emotional laborers experience significant suffering, and, thus, they are more likely to experience emotional exhaustion. Ashforth and Humphrey (1993) argued that when the surface behavior is performed while the disparity between the emotional state of the person and the needs of the organization exists, the worker experiences job exhaustion by dissatisfaction with his job and his own internal feelings. In addition, Brotheridge and Lee (2003) asserted that emotional exhaustion is caused by emotional incongruity. This suggests that emotional incongruity deepened by surface behavior causes emotional exhaustion.

Pugliesi (1999) found that both surface and internal actions negatively affect job satisfaction, while internal actions were less negative than surface activities. Ashforth and Humphrey (1993) suggested that the degree of exhaustion experienced while performing in-depth activities was low. In addition, Abraham (1998) and Morris and Feldman (1996) found that emotional labor is not necessarily negative, and in-depth behavior may be associated with positive outcomes because of positive feedback or self-fulfillment.

On the other hand, inner behavior refers to the effort to consciously modify the emotions of the employees according to their norms to express desirable behaviors (Hochschild, 1983), and emotional inconsistency between their actual behavior and how they actually feel is not large. It may not be easily confirmed that its effect on emotional exhaustion is smaller than the effect of the surface behavior, but the direction of the effect is opposite to the surface behavior. Because the concept of labor is included in the definition of emotional labor itself, internal action must also accompany the intended effort.

In the study of Kammeyer-Mueller et al. (2013), which meta-analyzes the leading and the inferring factors of surface and inner behavior, surface behavior showed a positive correlation with stress and burnout, while inner behavior was estimated to be positively correlated, but not statistically significant.

The influence of employees’ emotional exhaustion in service encounter is negative and extends from individuals to organizations. Emotional depletion and emotional exhaustion refer to a state of weakened sense of accomplishment that negatively affects one’s self, not just one’s emotions (Maslach and Jackson, 1981). Therefore, it is difficult to continuously treat people nicely when the person who is doing the work is emotionally depleted. In this context, Lee and Ashforth (1993) and Maslach et al. (1996) argue that job exhaustion affects job satisfaction and organizational commitment, reduce job satisfaction and increase absenteeism and turnover intentions.

There is also a direct negative relationship between emotional exhaustion and service performance (Wright and Cropanzano, 1998). Employees who have experienced emotional exhaustion will have difficulty making the same effort with their customers (Krishnan et al., 2002). Emotional employees are less likely to be considered genuinely thoughtful or pleasant by their customers, and experience limited patience when they respond to customers (Grandey, 2003). And Emotional Exhaustion affects counterproductive work behavior (Raman et al., 2016).

According to a study by Barger and Grandey (2006), emotions are known to have a contagion among organizational members as well as affecting job performance and job satisfaction of the parties. Emotional labor required as a role within an organization increases emotional exhaustion (Grandey, 2003), and emotional labor induces tension by emotional dysregulation and consumes emotional resources in the process of emotional labor (Hochschild, 1983; Wharton, 1993). In other words, emotional labor, which sells its emotions for pay, exhausts cognitive and emotional resources (Hochschild, 1983).

Previous researches regarding emotional labor have a common opinion that surface behaviors increase emotional exhaustion (Brotheridge and Grandey, 2002; Grandey, 2003), but there is a disagreement about the relationship between inner behavior and emotional exhaustion.

Grandey (2003) argued that both surface and inner behaviors can increase emotional exhaustion. Surface behaviors increase emotional exhaustion by increasing discrepancies between normative emotions and actual emotions, and inner actions can also consume considerable energy and effort to match emotions with normative emotions. In contrast, Brotheridge and Grandey (2002) argued that surface behavior increases emotional exhaustion, but internal action reduces emotional exhaustion. This is because employees can perform inner actions to minimize the state of emotional dissonance and achieve achievement through achieving high performance.

Grandey (2003) hypothesized that inner behaviors would increase emotional exhaustion, but research shows that internal behavior is not significant for emotional exhaustion. These results suggest that inner behavior minimizes emotional dissonance and affirms customer’s positive response. Based on the results of previous studies, this study hypothesized that each employee’s emotional expression and customer’s response would affect employee emotional exhaustion.

Hypothesis 2: Employees’ emotional expressions will affect emotional exhaustion.
[H2_1] Surface Acting → Emotional Exhaustion
[H2_2] Deep Acting → Emotional Exhaustion
[H2_3] Non-Verbal Expression → Emotional Exhaustion
[H2_4] Verbal Expression → Emotional Exhaustion
Hypothesis 3: Perception of Customer feedbacks will affect emotional exhaustion.
[H3_1] Customer Feedback (Positive) → Emotional Exhaustion
[H3_2] Customer Feedback (Negative) → Emotional Exhaustion

### Job Satisfaction

There have been various attempts to define Job Satisfaction. Hoppock (1935) defined job satisfaction as a combination of psychological, physiological, and environmental contexts of organizational members. Locke (1976) defined it as a pleasant and positive emotional state resulting from the evaluation of his or her job or job experience, and described it as being emotional and therefore being discovered and understood only by internal assumptions.

Job satisfaction is determined not only by the difference between the expectation recognized by the employees and the actual experience but also by the relationship between the organizational environment and personal characteristics such as the relationship between the supervisor and the employee. Job satisfaction is a personal response and can be viewed as an emotional response to a task or job (Bhuian and Mengue, 2002). It is known that emotions do not only affect individual performance and job satisfaction but also spread among the members of the organization (Barger and Grandey, 2006). In a recent study, job satisfaction refers to the state of emotions that the members feel in their subjective and relative view of the process of performing their duties and the results of their job performance, based on the individual’s values and beliefs (Park et al., 2011).

Job satisfaction is classified into various dimensions such as job, working environment, compensation, administration, position, and promotion depending on purpose and subject of research (Locke, 1976; Farber, 1991; Park et al., 2011). In terms of the society as a whole, the level of job satisfaction of the organizers is the basis for measuring the smooth operation and performance of the organization. Satisfaction with the job also affects the life outside the duty of the organizer; it has a positive side effect (Robbins, 1998). Employees’ satisfaction with their duties has a significant impact on the performance as their positive attitude toward the organization would have a great influence on the organization’s performance. Job satisfaction of the employees increases the quality of service and directly affects the evaluation of consumers, which is an important factor affecting repurchase (Brown and Peterson, 1993).

Job satisfaction is important because it contributes not only to personal happiness but also to the efficient operation and performance improvement of the organization. In addition to affecting positive mental health through satisfaction of work life on an individual level, job satisfaction is also known to have a direct relationship with exhaustion from work, low morale, and productivity.

In previous studies, emotional exhaustion has been reported as a major variable that negatively affects job satisfaction. The emotions of the employees facing the customers at the service contact point are related to the satisfaction of the customers, the emotions of the employees are related to the relationship with the customers, and they affect the job satisfaction (Macdonald and Sirianni, 1996; Kinnie et al., 1999). Also, a study by Park et al. (2011) on the manager of the retail industry showed that the manager’s emotional exhaustion had a negative effect on job satisfaction. Based on the previous findings, this study has established the following hypothesis.

Hypothesis 4: Employees’ emotional exhaustion will affect job satisfaction.

### Turnover Intentions

Price (2001) defined the turnover as the departure from the organizational status of the employees, which means disqualification as a member of the organization to which it belongs. The turnover intention, which is the stage immediately before the turnover, is used as an indicator to predict turnover. Employee turnover lowers other employees’ willingness and duties to remain in the firm. Further, departures of skilled labor workers would cause additional expenses in recruiting, hiring, and training of new employees. It will also have negative effects on the service quality of the organization. Therefore, higher turnover rate would negatively affect the organizations business performances (Mohsin et al., 2013). Therefore, there is an increasing tendency in interest and effort to decrease the turnover rate of the employees (Cho et al., 2009; Lee and Ryu, 2014).

Although there are various factors that cause the turnover of employees in the service industry, the one of the biggest problems is emotional exhaustion caused by emotional labor resulting from customer service work (Lee and Ashforth, 1996; Choi et al., 2012). Therefore, it is very important for the service organizations to pay close attention to job exhaustion as it would affect their turnover problems.

Based on the results of previous studies, this study has established the following hypothesis.

Hypothesis 5: Employees’ emotional exhaustion will affect turnover intention.
Hypothesis 6: Employees’ job satisfaction will affect turnover intention.

### Emotional Intelligence

Salovey and Mayer (1990) found that emotional intelligence enables us to observe and look at one’s emotions, to properly manage one’s emotions, to synchronize one’s own emotions, to empathize with other’s emotions, and to make good human relations. In other words, emotional intelligence is the ability to lead emotions productively and to synchronize oneself for their own goals in emotional situations. Therefore, emotional intelligence is associated with good job performance (Myers and DeWall, 2017).

In psychology, emotions are interpreted in the same way as emotional intelligence related to the know-hows involved in social situations and the successful management of oneself (Myers and DeWall, 2017). In the process of the perception of external stimuli, emotion has a significant influence on individual decision making according to age, gender, education, psychological state, family relation, economy, customs, religion, race (Prinz, 2007). It is also an interrelated skill related to the ability to create and approach emotions, the ability to understand emotions and emotional knowledge, and the ability to control emotions to promote emotional and intellectual growth, and is distinguished from general intelligence and personality (Mayer and Salovey, 1997; Wong and Law, 2002).

Goleman (1995) showed that emotional intelligence can synchronize individuals and keep oneself from frustration, control impulse and delay satisfaction, prevent emotional accidents and stressful accidents, and empathize with others. Abraham (1999) defined emotional intelligence as the ability to accurately assess and express emotions of oneself and others, the ability to regulate emotions, and the ability to use emotional knowledge to solve problems. Dulewicz and Higgs (2000) defined emotional intelligence as a concept that persuades and influences others by keeping it and motivating it, away from recognizing emotions.

Salovey and Mayer (1990) defined emotional intelligence as ’the ability to accurately perceive, express and express emotions, the ability to generate and use emotions to promote thinking, the ability to understand emotions and emotional knowledge, the ability to adjust emotions to promote development’. Mayer et al. (2016) added areas of reasoning and claimed that emotional intelligence contained the four major components: (a) the appraisal of emotions; (b) the understanding of emotions; (c) the management of emotions; and (d) the utilization of emotions.

Wong and Law (2002) extended the concept of emotional intelligence defined by Salovey and Mayer (1990), and refined the framework to categorize the sub-dimensions of emotional intelligence as perceiving, understanding, managing, and using emotions. Wong and Law (2002) also developed WLEIS questionnaire, and it has been used in subsequent studies such as Law et al. (2004) to verify reliability and validity.

It is necessary for the individual to recognize the reality and cause of his emotions, as well as to understand and empathize with emotions of others. The ability to express and manipulate controlled emotions is the behavior for problem solving and achievement. It has been found that the ability to self-manage emotions is a key factor in stressful situations and overall emotional management (Benson et al., 2012; Faguy, 2012; Rankin, 2013; Ivcevic and Brackett, 2014; Shanta and Gargiulo, 2014; Conley et al., 2015; Görgens-Ekermans et al., 2015; Ranjbar, 2015; Stelnicki et al., 2015; Fitzpatrick, 2016).

Previously, the effects of individual differences such as emotional intelligence or self-monitoring of emotional workers on emotional labor have been studied (Shin et al., 2008; Baeck et al., 2014). Emotional intelligence, the mental ability related to one’s emotional experiences (Mayer et al., 2016), was used to identify the cognitive determinants of emotional labor (Lee and Chelladurai, 2018). Also, the ability to perceive emotions, integrate emotion to facilitate thinking, understand, and manage emotions to promote personal growth are paramount to professional achievements and mental health and well-being (Mayer et al., 2016), and emotionally intelligent workers have positive attitudes toward their job and produce optimal performance (Pau and Sabri, 2015).

Emotional intelligence is known to be improved by learning, experience, and training (Gardner, 1995). People with high emotional intelligence tend to be sociable and well-perceived, and those with high emotional control tend to enjoy a high degree of interaction with others. Individuals with high emotional intelligence are better equipped to select effective emotional regulation strategies than those with low emotional intelligence (Wagstaff et al., 2012). Therefore, emotional intelligence should help minimize the negative impact of Surface Acting and enhance the positive effects of Deep Acting.

Laborde et al. (2016) noted that emotional intelligence was a marker of effective emotional regulation strategy. Lee and Chelladurai (2016) included both emotional labor and emotional intelligence in the study, and emotional intelligence was examined as a moderator between emotional labor and emotional exhaustion, rather than considering the direct relationship between emotional intelligence and emotional labor. Developed from these previous studies, Park (2018) examined the moderating effects of the sub-dimensions of Emotional Intelligence between Emotional Labor and Job Satisfaction. Perceiving Emotion and Understanding Emotion showed moderating effects while Managing Emotion and Using Emotion didn’t.

Although the importance of emotional intelligence receives more attention, there hasn’t been a comprehensive study to investigate the moderating effects of the sub-dimensions of emotional intelligence on Emotional Labor. Therefore, this study would use the Four-Branch Ability Model (Mayer and Salovey, 1997; Wong and Law, 2002; Mayer et al., 2016) to investigate the moderating effects of Emotional Intelligence.

#### Perceiving Emotion

The ability to accurately understand and express one’s emotions is used to better recognize and recognize one’s emotions. In other words, recognizing that emotion when it occurs is a major part of emotional intelligence.

#### Understanding Emotion

It is the core of emotional intelligence, and is the ability to understand and experience the emotions of others who are affected by such emotions. In addition, as a harmony of emotions, it is the source of self-awareness and altruism and the basis of moral judgment and action. Those with social competence carry out the functions of maintaining good interpersonal relationships, influencing others, successfully leading human relationships, and facilitating people.

#### Managing Emotion

Regulation of emotions forms the background of all accomplishments. The ability to control emotions for oneself is fast restoration ability in psychological frustration, restoration time from joy or anger to normal state, and self- restraint and control ability to control their emotions so that they do not become excited.

#### Using Emotion

The ability to utilize emotions to improve performance, and to control emotions in a positive and productive manner. A person with good self-synchronization is motivated by his inner desire for achievement, and is more efficient and productive in some work.

Based on the findings of previous studies, we propose that the previous hypotheses will show different results based on the level of emotional intelligence.

Hypothesis 7: Hypothesis 1 ∼ Hypothesis 6 will be different according to employee’s emotional intelligence (Perceiving emotion, Understanding emotion, Managing emotion, Using emotion).

## Materials and Methods

### Research Model

In previous studies, various external variables and parameters as well as moderator variables have been used to understand emotional labor and its related problems. Emotional Intelligence has been used in many studies in order to understand its effect on Emotional Labor at an individual level as well as at an organizational level (Miao et al., 2017). However, a comprehensive model to study the variables’ correlation and their overall effect on emotional labor has not yet been established. This study tries to understand the effects of emotion on emotional labor for both employees and customers at the service contact points, and test the role of emotional intelligence at each stage.

In order to accomplish this goal, a SEM would be used. SEM refers to a multivariate model that includes simultaneous equations, factor analysis, and multilevel models, for single and multi-group data. It is the combination of factor analysis and multiple regression analysis, and it is used to analyze structural relationship among variables. In the analysis using SEM, latent variables are created using multiple observed variables through factor analysis. Then, multiple regression analysis is performed on latent variables level.

Therefore, a SEM with Verbal and Non-Verbal Expressions, Customer Feedbacks, Surface Acting, and Deep Acting as observed variables, and Emotional Intelligence and its sub-dimensions as a moderator variable will be used to investigate the simultaneous relationship among Emotional Exhaustion, Job Satisfaction, Turnover Intentions, Verbal and Non-Verbal Expressions, Customer Feedbacks, Surface Acting, Deep Acting, and Emotional Intelligence.

### Variables

There exist various emotional labor related problems including Employee Well-being and Organizational Well-being (Diefendorff et al., 2011; Grandey and Gabriel, 2015), Self-actualization through the interaction, Job Commitment, Professional Efficacy, Turnover Intentions (Quinones et al., 2016), Job Stressor (Mauno et al., 2017), Job Burnout (Chan, 2006; Zhao et al., 2018), Emotional Burden in the Interaction (Hujala and Oksman, 2018), and Work-life balance and Job Satisfaction (Hofmann and Stokburger-Sauer, 2017).

External Factors that are related to emotional labor can be divided into person characteristics related factors and event characteristics related factors. Person characteristics related factor means person-job congruence, and event characteristics related factor means emotion-goal congruence (Grandey and Gabriel, 2015). Person characteristics refer to personality traits, work motives, and emotional abilities, while event characteristics refer to moods, emotions, and customer mistreatment. Emotional Intelligence (Lee and Chelladurai, 2018), Frequency of Emotional Display, Attentiveness to Required Display Rules, Variety of Emotions Required to be expressed, Emotional Dissonance (Morris and Feldman, 1996; Hayyat et al., 2017; Serebrenik, 2017) have also been used. In addition to these factors, two other variables should be taken into consideration in order to fully understand emotional labor: Emotional Regulations and Emotional Intelligence. The former refers to external factors that dictate how employees should express their emotions, and the latter refers to employees’ ability to control their emotions and empathize with customers (Grandey and Gabriel, 2015).

Various control variables have been used in previous studies. Kim et al. (2017) used perceived supports from supervisors, co-workers, and organization as control variables. There are also relational factors and contextual factors (Grandey and Gabriel, 2015). Relational factors include emotional traits/abilities, identification/values, and relational power/intimacy, and contextual factors include job status/autonomy, financial reward, and social support. Emotional Intelligence (Lee and Chelladurai, 2016) and Emotion Regulation Self-Efficacy (Deng et al., 2017) have also been used.

This study will follow Grandey and Gabriel (2015) and use Emotion Regulation (Surface Acting and Deep Acting) and Emotion Performance (Verbal and Non-Verbal Communication) at the same time as factors to analyze Emotional Labor. This study will focus on the structural relationship among Emotional Exhaustion, Job Satisfaction, and Turnover Intention with four factors mentioned above and Emotional Intelligence as a moderator variable.

### Hypotheses

Hypothesis 1: Employees’ emotional expressions will affect perception of customer feedback.
[H1_a1] Surface Acting → Customer Feedback (Positive)
[H1_a2] Deep Acting → Customer Feedback (Positive)
[H1_a3] Non-Verbal Expression → Customer Feedback (Positive)
[H1_a4] Verbal Expression → Customer Feedback (Positive)
[H1_b1] Surface Acting → Customer Feedback (Negative)
[H1_b2] Deep Acting → Customer Feedback (Negative)
[H1_b3] Non-Verbal Expression → Customer Feedback (Negative)
[H1_b4] Verbal Expression → Customer Feedback (Negative)
Hypothesis 2: Employees’ emotional expressions will affect emotional exhaustion.

[H2_1] Surface Acting → Emotional Exhaustion
[H2_2] Deep Acting → Emotional Exhaustion
[H2_3] Non-Verbal Expression → Emotional Exhaustion
[H2_4] Verbal Expression → Emotional Exhaustion
Hypothesis 3: Perception of Customer feedbacks will affect emotional exhaustion.
[H3_1] Customer Feedback (Positive) → Emotional Exhaustion
[H3_2] Customer Feedback (Negative) → Emotional Exhaustion

Hypothesis 4: Employees’ emotional exhaustion will affect job satisfaction.

Hypothesis 5: Employees’ emotional exhaustion will affect turnover intention.

Hypothesis 6: Employees’ job satisfaction will affect turnover intention.

Hypothesis 7: Hypothesis 1 ∼ Hypothesis 6 will be affected by employee’s emotional intelligence (Perceiving Emotion, Understanding Emotion, Managing Emotion, Using Emotion).

### Sample and Measures

The survey has been conducted to collect data on emotional labor and emotional intelligence at the service contact points. Survey questionnaires were created with enhancements and supplemental questions utilizing previous studies. The 7-point response format ranged from 1 (strongly disagree) to 7 (strongly agree). A structured questionnaire was used to collect the data from the employees of the service industry using internet survey. Five hundred and seventy nine data was collected through convenience sampling and 577 valid data were used in the analysis. Two invalid data were disregarded.

Industries that are included in this study are Wholesale or Retail Stores (19.8%), Restaurants (19.4%), Telecommunication (14.7%), Education Service (10.6%), and others (35.5%). Mean age of participants of the survey is 34.7 (SD = 10.7). 59.1% of participants were male and 40.9% were female.

Socio-Demographic Characteristics of participants are provided in Table 1 and the list of Measured Variables are provided in Table 2.

**Table 1:** Respondents’ socio-demographic characteristics.

Variable	Group	N (%)
Gender	Male	341(59.1)
	Female	236(40.9)
	Total	577(100.0)
Age	Less than 30 years	200(34.9)
	30∼39years	237(41.4)
	40∼49 years	77(13.4)
	50 years or more	59(10.3)
	Total	573(100.0)
	Mean(*SD*)	34.7(10.7)
Annual Income	Less than $30,000	183(31.7)
	$30,000∼$50,000	157(27.2)
	$50,000∼$70,000	104(18.0)
	$70,000 or more	133(23.1)
	Total	577(100.0)
Education	Graduated high school	111(19.2)
	Graduated college	299(51.8)
	Post- graduate study without degree	37(6.4)
	Post-graduate degree	130(22.5)
	Total	577(100.0)
Marital status	Married	311(53.9)
	Single	232(40.2)
	Separated/Divorced/Widowed	34(5.9)
	Total	577(100.0)
Position	Ordinary employee	312(54.1)
	Manager	225(39.0)
	Director/CEO	40(6.9)
	Total	577(100.0)
Employment type	Regular job	505(87.5)
	Temporary job	43(7.5)
	Freelance work	29(5.0)
	Total	577(100.0)
Business type	Bank/Insurance	81(14.0)
	Whole sale or retail(department store/mart/store)	114(19.8)
	Transportation (bus/airplane/ship)	23(4.0)
	Tele communication (information service)	85(14.7)
	Restaurant	112(19.4)
	Education service	61(10.6)
	Leisure(art/sport/culture)	22(3.8)
	Etc.	79(13.7)
	Total	577(100.0)

**Table 2:** Measurements of variables.

Construct		Measurement items	Sources
Surface Acting		(1) I put on an acting when interacting with customers.	Brotheridge and Lee, 2003; Grandey et al., 2005; Üzümcü et al., 2017
		(2) I show feelings to customers that are different from what I feel inside.	
		(3) I fake the emotions I show when dealing with customers.	
Deep Acting		(1) I make an effort to actually feel the emotions that I need to display toward others.	Brotheridge and Lee, 2003; Kipfelsberger et al., 2016; Üzümcü et al., 2017
		(2) I try to feel the emotions that I’m expressing to my customers.	
		(3) I work at developing the feelings inside of me that I need to show to customers.	
Non-Verbal Expression		(1) I smile kindly to the customer.	Burgoon et al., 1990; Gabbott and Hogg, 2000; Sundaram and Webster, 2000
		(2) I keep eye contact with customers appropriately when talking.	
		(3) I greet my customers with familiarity.	
		(4) I serve with a big smile.	
Verbal Expression		(1) I talk to customers in the right tone.	
		(2) I talk to customers in a gentle tone.	
		(3) I talk to customers in a reasonable voice without being noisy.	
Perception of Customer Feedback Positive		(1) My customer seemed satisfied.	Watson et al., 1988; Mano and Oliver, 1993; Richins, 1997; Bagozzi et al., 1999; Ryu and Jang, 2007
		(2) My customer seemed happy.	
		(3) My customer seemed good.	
		(4) My customer seemed pleased.	
Perception of Customer Feedback Negative		(1) My customer seemed angry.	
		(2) My customer seemed hostile to me.	
		(3) My customer seemed nervous.	
		(4) My customer felt unhappy.	
Emotional Exhaustion		(1) I’m exhausted from work.	Maslach and Jackson, 1981; Wharton, 1993; Quinones et al., 2017
		(2) I feel frustrated about my job.	
		(3) I feel tired when I finish work.	
Job Satisfaction		(1) My company gives equal opportunity to get a promotion based on their hard work.	Brown and Peterson, 1993; Spector, 1997
		(2) My career helps me develop and improve my skills.	
		(3) My current salary is fair in the light of what I do.	
		(4) My boss takes suggestions and complaints with sincerity.	
		(5) I am satisfied with the company’s policy.	
Turnover Intentions		(1) Sometimes, I’d like to work for another company.	Mobley, 1977; Cropanzano et al., 1993; Quinones et al., 2017
		(2) If reemployment is guaranteed and I am financially stable, I will quit this job.	
		(3) I’d like to quit this job if I could.	
Emotional Intelligence	Perceiving emotion	(1) I usually know why I have such feelings.	Wong and Law, 2002
		(2) I understand my feelings well.	
		(3) I understand what I feel.	
		(4) I always know whether I am happy or not.	
	Understanding emotion	(1) I can usually understand people’s feelings through their actions.	
		(2) I’m good at understanding other people’s emotions.	
		(3) I understand other people’s feelings well.	
		(4) I am sensitive to the feelings of others.	
	Managing emotion	(1) I can control my anger.	
		(2) I can calm down myself quickly, even when I’m angry.	
		(3) I have the ability to control my emotions (sensibility).	
		(4) I have the ability to control my feelings.	
	Using emotion	(1) I always do my best to achieve my goals.	
		(2) I believe myself that I am a competent person.	
		(3) I am a self-motivated person.	
		(4) I encourage myself to do my best.	

### Overview of Statistical Analyses

Data analysis of this study utilized SPSS 24 and AMOS 18 programs. The hypotheses were tested using frequency analysis and confirmatory factor analysis, structural equation, and statistical analysis method of group comparison.

Firstly, descriptive statistics and the reliability of the variables were computed using SPSS 24.0. Then a structural equation modeling technique, which is available through AMOS 18.0, was used to test both the measurement and structural models. Confirmatory factor analysis (CFA) was conducted on the latent variables to check convergent and discriminant validity of the variables, and then the proposed structural relationship was tested by assessing structural coefficients of the relationship among the constructs in the research model. CFA is used to study the relationships between a set of observed variables and a set of continuous latent variables. The measurement model for CFA is a multivariate regression model that describes the relationships between a set of observed dependent variables and a set of continuous latent variables.

Further, the moderating effect of emotional intelligence was tested. Multisample confirmatory factors analysis (MCFA) allowed us to determine if components of the structural model were equal across different groups (Byrne, 2001). Moderating effects of Emotional Intelligence that comprises four factors (Perceiving Emotion, Understanding Emotion, Managing Emotion, and Using Emotion) were tested using Multi-Group Structural Equation Model (MSEM).

Cross Validation was performed using Multi-Sample Confirmatory Factor Analysis (MCFA). This method allowed us to conduct analyses at multiple levels at the same time in a single analysis. By decomposing the variance of variables into their between-group and within-group components, MSEM accounts for the fact that relationships might be different on the between-group and the within-group levels. Thus, multilevel mediation analyses with MSEM are less prone to biases than other techniques of multilevel mediation analysis (Zhang et al., 2009).

Confirmatory factor analysis on the measurement model was performed using AMOS 18.0. Goodness-of-Fit Test on the model using maximum likelihood estimation showed χ^2^= 844.01, df = 426, *p*-value = 0.00, GFI = 0.91, RMSEA = 0.04, NFI = 0.94, RFI = 0.93, IFI = 0.97, and CFI = 0.97, indicating that the model shows good fit indices of measured variables on overall latent factor. Further, standardized factor loading is greater than 0.6, indicating that it is statistically significant. Detailed result of CFA is provided in Table 3.

**Table 3:** Reliability and validity tests.

Variable	Indicator	Estimate	*t*-value	SMC	Cronbach’s *a*	AVE	CR
Surface Acting	SA_1	1.00	–	0.65	0.85	0.65	0.85
	SA_2	0.98	19.53	0.65			
	SA_3	1.03	19.53	0.65			
Deep Acting	DA_1	1.00	–	0.69	0.85	0.65	0.85
	DA_2	0.97	21.22	0.67			
	DA_3	0.92	19.89	0.60			
Non-Verbal Expression	NVE_1	1.00	23.46	0.72	0.87	0.64	0.88
	NVE_2	1.00	–	0.66			
	NVE_3	0.92	20.74	0.61			
	NVE_4	0.93	20.55	0.58			
Verbal Expression	VE_1	1.00	–	0.72	0.84	0.64	0.84
	VE_2	0.89	19.40	0.52			
	VE_3	0.98	23.07	0.67			
Customer Feedback Positive	CFP_1	1.00	–	0.58	0.88	0.65	0.88
	CFP_2	1.05	20.40	0.70			
	CFP_3	0.99	19.74	0.66			
	CFP_4	1.03	19.77	0.66			
Customer Feedback Negative	CFN_1	1.00	–	0.79	0.93	0.76	0.93
	CFN_2	1.02	30.80	0.80			
	CFN_3	0.94	27.35	0.71			
	CFN_4	1.02	29.64	0.77			
Emotional Exhaustion	EE_1	1.00	–	0.62	0.85	0.62	0.83
	EE_2	1.15	22.07	0.75			
	EE_3	0.85	21.65	0.49			
Job Satisfaction	JS_1	1.15	20.52	0.71	0.89	0.62	0.89
	JS_2	1.13	19.60	0.66			
	JS_3	1.06	17.22	0.52			
	JS_4	1.00	–	0.56			
	JS_5	1.08	19.80	0.67			
Turnover Intentions	TI_1	1.00	–	0.78	0.92	0.79	0.92
	TI_2	0.98	29.08	0.76			
	TI_3	1.06	31.96	0.85			

*Goodness-of-fit: x^2^ = 844.01, df = 426, p = 0.00, GFI = 0.91, RMSEA = 0.04, NFI = 0.94, RFI = 0.93, IFI = 0.97, TLI = 0.96, CFI = 0.97.*

Construct Reliability (CR) and Average Variance Extracted (AVE) of each variable satisfy general standards (CR > 0.7, AVE > 0.5) and therefore have Convergent Validity (Bagozzi, 1988).

Discriminant Validity can be tested by using AVE-SE comparison (Fornell and Larcker, 1981) using error-adjusted inter-construct correlation derived from CFA. Results provided in Table 4 show that SE does not exceed AVE and therefore indicating that all constructs showed sufficient discriminant validity.

**Table 4:** Construct means, standard deviations, and correlations.

	SA	DA	NVE	VE	CFP	CFN	EE	JS	TI
SA	1.00								
DA	0.05	1.00							
NVE	0.13	0.52	1.00						
VE	0.11	0.46	0.80	1.00					
CFP	0.01	0.45	0.54	0.47	1.00				
CFN	0.37	0.07	−0.19	−0.19	−0.16	1.00			
EE	0.49	0.04	−0.07	−0.07	−0.09	0.56	1.00		
JS	−0.02	0.60	0.47	0.36	0.52	0.01	−0.20	1.00	
TI	0.44	−0.09	−0.12	−0.11	−0.14	0.45	0.74	−0.31	1.00
Means	4.76	5.09	5.62	5.70	5.35	3.27	4.20	5.09	4.13
*SD*	1.45	1.31	1.14	1.10	1.19	1.72	1.64	1.31	1.90

*SA, Surface Acting; DA, Deep Acting; NVE, Non-Verbal Expression; VE, Verbal Expression; CFP, Perception of Customer Feedback (Positive); CFN, Perception of Customer Feedback (Negative); EE, Emotional Exhaustion; JS, Job Satisfaction; TI, Turnover Intentions.*

Then SEM was analyzed as validity and reliability of the variables have been confirmed.

## Results

### Structural Models

The model showed good fit and all indices satisfy the acceptance criteria (χ^2^= 1282.10, df = 439, *p*-value = 0.00, GFI = 0.88, RMSEA = 0.06, NFI = 0.90, RFI = 0.89, IFI = 0.93, TLI = 0.93, CFI = 0.93).

Test results showed that Deep Acting (Estimate = 0.22, *t*-value = 3.95, *p*-value = 0.00) and Non-Verbal Communication (Estimate = 0.66, *t*-value = 3.11, *p*-value = 0.00) have statistically significant effects on Perception of Customer Feedback (Positive) while Surface Acting and Verbal Expression failed to show significant effects. Therefore, [H1_a2] and [H1_a3] were supported while [H1_a1] and [H1_a4] were not supported.

Surface Acting (Estimate = 0.47, *t*-value = 10.20, *p*-value = 0.00), Deep Acting (Estimate = 0.33, *t*-value = 5.65, *p*-value = 0.00), and Non-Verbal Communication (Estimate = −0.51, *t*-value = −2.39, *p*-value = 0.02) all showed statistically significant effects on Perception of Customer Feedback (Negative). Only Verbal Communication failed to show statistically significant effect on Perception of Customer Feedback (Negative). As a result, [H1_b1], [H1_b2], and [H1_b3] were supported while [H1_b4] was not supported.

When the effects of variables on Emotional Exhaustion were tested, only Surface Acting showed significant effect (Estimate = 0.40, *t*-value = 8.24, *p*-value = 0.00). Deep Acting (Estimate = 0.00, *t*-value = 0.01, *p*-value = 0.63), Non-Verbal Communication (Estimate = −0.09, *t*-value = −0.48, *p*-value = 0.99), and Verbal Communication (Estimate = 0.01, *t*-value = 0.05, *p*-value = 0.96) all failed to show significant effects and, thus, [H2_2], [H2_3], and [H2_4] were not supported while [H2_1] was supported.

Test results support that customers’ reactions and feedbacks affect employees’ emotional exhaustion as well. Although Perception of Customer Feedback (Positive) didn’t show significance (Estimate = −0.01, *t*-value = −0.14, *p*-value = 0.89) and, therefore, [H3_1] was not supported, Perception of Customer Feedback (Negative) showed significance (Estimate = 0.47, *t*-value = 9.60, *p*-value = 0.00) for [H3_2] to be supported.

Employees’ emotional exhaustion showed statistically significant effects on both Job Satisfaction (Estimate = −0.23, *t*-value = −4.70, *p*-value = 0.00) and Turnover Intention (Estimate = 0.79, *t*-value = 18.18, *p*-value = 0.00) and, therefore, both [H4] and [H5] were supported.

Finally, Job Satisfaction does have a significant effect (Estimate = −0.18, *t*-value = −5.63, *p*-value = 0.00) on Turnover Intention and therefore [H6] was supported.

**FIGURE 1:**
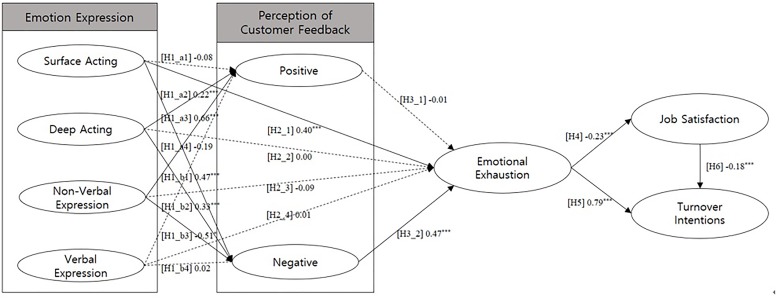
Research results. Solid lines represent significant relationships; dotted lines represent non-significant relationships.

Detailed results of the test on each hypothesis are provided in Table 5 and Figure 1.

**Table 5:** Structural models results.

Structural Path		Estimate	*t-value*	*p-value*
H1_a1 Surface Acting	→ Customer Feedback (Positive)	−0.08	−1.96	0.05
H1_a2 Deep Acting	→ Customer Feedback (Positive)	0.22	3.95^∗∗∗^	0.00
H1_a3 Non-Verbal Expression	→ Customer Feedback (Positive)	0.66	3.11^∗∗^	0.00
H1_a4 Verbal Expression	→ Customer Feedback (Positive)	−0.19	−0.96	0.34
H1_b1 Surface Acting	→ Customer Feedback (Negative)	0.47	10.20^∗∗∗^	0.00
H1_b2 Deep Acting	→ Customer Feedback (Negative)	0.33	5.65^∗∗∗^	0.00
H1_b3 Non-Verbal Expression	→ Customer Feedback (Negative)	−0.51	−2.39^∗^	0.02
H1_b4 Verbal Expression	→ Customer Feedback (Negative)	0.02	0.08	0.94
H2_1 Surface Acting	→ Emotional Exhaustion	0.40	8.24^∗∗∗^	0.00
H2_2 Deep Acting	→ Emotional Exhaustion	0.00	0.01	0.63
H2_3 Non-Verbal Expression	→ Emotional Exhaustion	−0.09	−0.48	0.99
H2_4 Verbal Expression	→ Emotional Exhaustion	0.01	0.05	0.96
H3_1 Customer Feedback (Positive)	→ Emotional Exhaustion	−0.01	−0.14	0.89
H3_2 Customer Feedback (Negative)	→ Emotional Exhaustion	0.47	9.60^∗∗∗^	0.00
H4 Emotional Exhaustion	→ Job Satisfaction	−0.23	−4.70^∗∗∗^	0.00
H5 Emotional Exhaustion	→ Turnover Intention	0.79	18.18^∗∗∗^	0.00
H6 Job Satisfaction	→ Turnover Intention	−0.18	−5.63^∗∗∗^	0.00

*Goodness-of-fit: x^2^ = 1282.10, df = 439, p = 0.00, GFI = 0.88, RMSEA = 0.06, NFI = 0.90, RFI = 0.89, IFI = 0.93, TLI = 0.93, CFI = 0.93.*

### Moderating Effects of Emotional Intelligence

Moderating effects of Emotional Intelligence that comprises four factors (Perceiving Emotion, Understanding Emotion, Managing Emotion, and Using Emotion) were tested using MSEM. Cross Validation was performed using MCFA, then multiple group comparison analysis was conducted in order to test overall moderating effect of emotional intelligence level compared to Unconstrained Model (Table 6).

**Table 6:** Moderating effect of Emotional Intelligence.

Hypothesis	Perceiving emotion			Understanding emotion			Managing emotion			Using emotion		
	Low(296)	High(281)	Critical Ration	Low(304)	High(273)	Critical Ration	Low(283)	High(294)	Critical Ration	Low(305)	High(272)	Critical Ration
	Coefficient (*t*-Value)	Coefficient (*t*-Value)		Coefficient (*t*-Value)	Coefficient (*t*-Value)		Coefficient (*t*-Value)	Coefficient (*t*-Value)		Coefficient (*t*-Value)	Coefficient (*t*-Value)	
H1_a1	0.04 (0.38)	−0.13 (−2.04)^∗^	−1.21	−0.03 (−0.37)	−0.10 (−1.54)	−0.44	−0.02 (−0.30)	−0.12 (−2.00)^∗^	−0.82	0.12 (1.24)	−0.11 (−1.94)	−1.97
H1_a2	0.25 (2.46)^∗^	0.26 (3.43)^∗∗∗^	−0.12	0.23 (2.58)^∗^	0.18 (1.98)^∗^	−0.28	0.17 (1.92)	0.24 (2.85)^∗∗^	0.53	0.14 (1.45)	0.27 (3.36)^∗∗∗^	1.24
H1_a3	0.73 (2.05)^∗^	0.33 (1.86)	−0.16	0.61 (2.13)^∗^	0.61 (1.48)	0.57	0.72 (2.73)^∗∗^	0.46 (1.44)	0.08	0.67 (2.33)^∗^	0.42 (1.93)	0.48
H1_a4	−0.31 (−0.91)	−0.07 (−0.45)	0.41	−0.24 (−0.95)	−0.24 (−0.62)	−0.23	−0.21 (−0.87)	−0.17 (−0.57)	−0.16	−0.22 (−0.81)	−0.10 (−0.51)	0.07
H1_b1	0.45 (4.00)^∗∗∗^	0.48 (7.95)^∗∗∗^	−0.14	0.48 (4.77)^∗∗∗^	0.47 (7.75)^∗∗∗^	0.44	0.44 (4.97)^∗∗∗^	0.49 (8.44)^∗∗∗^	1.41	0.51 (4.48)^∗∗∗^	0.45 (7.48)^∗∗∗^	−0.86
H1_b2	0.67 (5.20)^∗∗∗^	0.19 (2.71)^∗∗^	−2.95^∗∗^	0.54 (5.48)^∗∗∗^	0.17 (2.12)^∗^	−1.86	0.57 (5.48)^∗∗∗^	0.16 (2.05)^∗^	−2.26^∗^	0.54 (4.93)^∗∗∗^	0.18 (2.33)^∗^	−2.23^∗^
H1_b3	−0.97 (−2.40)^∗^	−0.19 (−1.18)	0.86	−0.83 (−2.69)^∗∗^	−0.16 (−0.44)	0.51	−0.74 (−2.68)^∗∗^	−0.20 (−0.69)	0.33	−0.88 (−2.77)^∗∗^	−0.08 (−0.39)	1.13
H1_b4	0.12 (0.31)	−0.06 (−0.38)	−0.48	0.19 (0.73)	−0.12 (−0.37)	−0.58	−0.06 (−0.22)	−0.06 (−0.22)	−0.13	0.14 (0.47)	−0.20 (−1.03)	−1.11
H2_1	0.64 (4.85)^∗∗∗^	0.31 (5.20)^∗∗∗^	−2.30^∗^	0.60 (5.13)^∗∗∗^	0.31 (5.09)^∗∗∗^	−1.67	0.52 (4.90)^∗∗∗^	0.31 (5.55)^∗∗∗^	−1.34	0.58 (4.58)^∗∗∗^	0.32 (5.51)^∗∗∗^	−2.09^∗^
H2_2	0.24 (1.64)	−0.03 (−0.55)	−1.70	0.13 (1.19)	−0.04 (−0.54)	−1.22	0.07 (0.60)	−0.05 (−0.75)	−0.93	−0.05 (−0.49)	0.03 (0.41)	0.64
H2_3	−0.55 (−1.36)	0.02 (0.16)	1.09	−0.56 (−1.78)	0.22 (0.68)	1.25	−0.41 (−1.36)	0.21 (0.83)	1.36	−0.11 (−0.37)	−0.13 (−0.74)	−0.41
H2_4	0.20 (0.60)	−0.15 (−1.16)	−1.22	0.31 (1.27)	−0.26 (−0.85)	−1.21	0.32 (1.30)	−0.38 (−1.64)	−2.02^∗^	0.10 (0.40)	0.01 (0.08)	−0.17
H3_1	0.06 (0.61)	−0.05 (−0.87)	−1.02	0.09 (1.15)	−0.08 (−1.36)	−1.78	0.05 (0.56)	−0.05 (−0.93)	−1.02	−0.02 (−0.18)	−0.01 (−0.10)	0.08
H3_2	0.32 (3.00)^∗∗^	0.50 (8.28)^∗∗∗^	1.88	0.36 (3.97)^∗∗∗^	0.52 (8.34)^∗∗∗^	1.70	0.38 (3.80)^∗∗∗^	0.50 (8.66)^∗∗∗^	0.78	0.38 (3.93)^∗∗∗^	0.50 (8.25)^∗∗∗^	1.17
H4	0.13 (1.77)	−0.31 (−4.73)^∗∗∗^	−4.19^∗∗∗^	0.04 (0.62)	−0.34 (−5.07)^∗∗∗^	−3.60^∗∗∗^	−0.09 (−1.27)	−0.30 (−4.66)^∗∗∗^	−1.46	0.19 (2.61)^∗∗^	−0.36 (−5.37)^∗∗∗^	−5.44^∗∗∗^
H5	0.83 (10.11)^∗∗∗^	0.77 (14.33)^∗∗∗^	−1.27	0.80 (10.11)^∗∗∗^	0.80 (15.19)^∗∗∗^	−0.28	0.74 (9.51)^∗∗∗^	0.83 (15.86)^∗∗∗^	0.20	0.89 (10.77)^∗∗∗^	0.78 (13.83)^∗∗∗^	−0.80
H6	−0.28 (−4.82)^∗∗∗^	−0.20 (−4.78)^∗∗∗^	0.50	−0.30 (−5.27)^∗∗∗^	−0.15 (−3.68)^∗∗∗^	1.56	−0.33 (−5.84)^∗∗∗^	−0.11 (−2.73)^∗∗^	2.50^∗^	−0.30 (−5.35)^∗∗∗^	−0.16 (−3.70)^∗∗∗^	1.43

*^∗^p < 0.05, ^∗∗^p < 0.01, ^∗∗∗^p < 0.001. H1_a1 Surface Acting → Customer Feedback (Positive) H1_a2 Deep Acting → Customer Feedback (Positive) H1_a3 Non-Verbal Expression → Customer Feedback (Positive) H1_a4 Verbal Expression → Customer Feedback (Positive) H1_b1 Surface Acting → Customer Feedback (Negative) H1_b2 Deep Acting → Customer Feedback (Negative) H1_b3 Non-Verbal Expression → Customer Feedback (Negative) H1_b4 Verbal Expression → Customer Feedback (Negative) H2_1 Surface Acting → Emotional Exhaustion H2_2 Deep Acting → Emotional Exhaustion H2_3 Non-Verbal Expression → Emotional Exhaustion H2_4 Verbal Expression → Emotional Exhaustion H3_1 Customer Feedback (Positive) → Emotional Exhaustion H3_2 Customer Feedback (Negative) → Emotional Exhaustion H4 Emotional Exhaustion → Job Satisfaction H5 Emotional Exhaustion → Turnover Intention H6 Job Satisfaction → Turnover Intention*

The analysis of MSEM of Perceiving Emotion showed that the paths from Deep Acting to Perception of Customer Feedback (Negative) (Critical Ration = −2.95, *p*-value < 0.01), Surface Acting to Emotional Exhaustion (Critical Ration = −2.30, *p*-value < 0.05), and Emotional Exhaustion to Job Satisfaction (Critical Ration = −4.19, *p*-value < 0.00) were significant. This result could be interpreted as that the effects of Deep Acting on Perception of Customer Feedback (Negative) is bigger for the group with Low Perceiving Emotion, while the effect of Surface Acting on Emotional Exhaustion and the effect of Emotional Exhaustion on Job Satisfaction are greater for the group with High Emotional Intelligence.

The analysis on MSEM of Understanding Emotion showed that the path from Emotional Exhaustion to Job Satisfaction (Critical Ration = −3.60, *p*-value < 0.00) is significant. The result shows that the effect of Emotional Exhaustion on Job Satisfaction is greater for a group with High Emotional Intelligence.

The analysis on MSEM of Managing Emotion showed that the paths from Deep Acting to Perception of Customer Feedback (Negative) (Critical Ration = −2.26, *p*-value < 0.05), Verbal Expression to Emotional Exhaustion (Critical Ration = −2.02, *p*-value < 0.05), and Job Satisfaction to Turnover Intention (Critical Ration = 2.50, *p*-value < 0.05) were significant. The effects of Deep Acting on Perception of Customer Feedback (Negative) and Job Satisfaction on Turnover Intention were greater for a group with Low Emotional Intelligence, while the effect of Verbal Expression on Emotional Exhaustion was greater for a group with High Emotional Intelligence.

The analysis on MSEM of Using Emotion showed that the paths from Deep Acting to Perception of Customer Feedback (Negative) (Critical Ration = −2.23, *p*-value < 0.05), Surface Acting to Emotional Exhaustion (Critical Ration = −2.09, *p*-value < 0.05), and Emotional Exhaustion to Job Satisfaction (Critical Ration = −5.44, *p*-value < 0.00) were significant. The effect of Deep Acting on Perception of Customer Feedback (Negative) is greater for a group with Low Emotional Intelligence, while the effects of Surface Acting on Emotional Exhaustion and Emotional Exhaustion on Job Satisfaction are greater for a group with High Emotional Intelligence.

## Discussion

In order to provide solutions to the problems regarding Emotional Labor, especially in service industry, two main approaches were used in this study. Firstly, we looked at Emotional Labor in the perspective of Emotional Expression regulated by organizational norms. Then we looked at Emotional Intelligence which is individuals’ ability to understand and control his/her own emotions. As looking into these two perspectives simultaneously could provide more in-depth understanding of the nature of Emotional Labor, a SEM with various emotional expressions of the employees and the perception of customer feedbacks was tested in order to test how these variables are related to and affecting emotional exhaustion, job satisfaction, and turnover intention.

The results show that both Deep Acting and Surface Acting are valid influential factors to recognize customer’s reactions. Deep Acting of employees is a valid factor to recognize customers’ positive and negative reactions. It suggests that employees’ effort to understand or experience their true feelings is the behavior intending to understand customers’ emotion in service encounter. Also, the employees’ Surface Acting is a valid factor to recognize customers’ negative reactions. In other words, employees’ behavior to hide their true feelings and express their emotions according to organizations’ regulations and norms affect employees’ ability to recognize customers’ negative reactions.

Non-Verbal Expression was shown to be a factor that increases Perception of Customer Feedback (Positive) and reduces Perception of Customer Feedback (Negative). This result is consistent with previous findings regarding the characteristics of Non-Verbal Expression. Body language can convey interest, intimacy, benevolent feelings, and trust to customers (Mehrabian and Williams, 1969; Burgoon et al., 1990), and non-verbal communication narrows the psychological distance between employees and customers, increasing positive emotions (Sundaram and Webster, 2000).

The effects of Non-Verbal Communication might vary according to the characteristics of services provided and of customers. Therefore, more detailed studies on the guideline of Non-Verbal Communication that incorporates the characteristics of the service and the customers would be necessary. Further, as online services grow, in-depth study on non-verbal online communication might provide meaningful insights as well.

In addition, it was shown that customers’ negative reaction influences the way Surface Acting leads to Emotional Exhaustion. This result supports the previous findings that the employees’ Surface Acting negatively affects employees themselves while Deep Acting positively affects employees. Therefore, more systematic and effective approaches that will enable employees to act and behave based on voluntary Deep Acting at the service contact points should be established rather than coercing employees into unconditional Surface Acting in order to find solutions to the problems related to Emotional Labor.

Emotional Exhaustion caused by Emotional Labor and Perception of Customer Feedback lowers employees’ Job Satisfaction and increases Turnover Intention, while Job Satisfaction lowers Turnover Intention, which, again, support previous findings. Therefore, in order to lower Turnover Intention, organizations should find ways to increase Job Satisfaction and reduce factors that lead to Emotional Exhaustion.

This study also tested four factors of Emotional Intelligence in order to verify its moderating effects. The results show that it affects Emotional Exhaustion and Job Satisfaction, and hence, Turnover Intention.

The results of the analysis on Perceiving Emotion show that there exists a difference between the effect of Deep Acting on Perception of Customer Feedback (Negative) and the effect of Surface Acting on Emotional Exhaustion. This result suggests that an employee with better understanding of his/her own emotions experience greater negative effect when there is a discrepancy between what they feel and how they should act. Therefore, organizations should collect employees’ opinions prior to establishing organizational guidelines of employee behaviors. Behavioral guidelines that incorporate employees’ opinions would help reduce Emotional Exhaustion caused by Emotional Labor.

The result of the analysis on Understanding Emotion also suggests that the effect of Emotional Exhaustion on Job Satisfaction depends on the level of Emotional Intelligence. An employee with higher Emotional Intelligence experiences a greater effect on Job Satisfaction from Emotional Exhaustion.

The analysis on Managing Emotion suggests a similar result. It shows that the effect of Deep Acting on Perception of Customer Feedback (Negative) and the effect of Job Satisfaction on Turnover Intention vary depending on the employees’ Emotional Intelligence. This result could be interpreted as that an employee with a high Emotional Intelligence is more likely to recover from psychological frustration and emotional depletion and therefore would have lower Turnover Intention.

Lastly, the analysis on Using Emotion showed that the effect of Deep Acting on Perception of Customer Feedback (Negative), the effect of Surface Acting on Emotional Exhaustion, and the effect of Emotional Exhaustion on Job Satisfaction vary depending on Emotional Intelligence. This result confirms that the ability to utilize his/her own emotions would enhance Job Performance of employees.

### Contributions and Implications

There are two main perspectives of this research. The first is to look for solutions through the governing principles that control workers’ emotional expressions. The second is to look for solutions through individually controlled emotional intelligence. Utilizing these two perspectives that are independent yet interrelated, the more thorough and effective solutions to the problems regarding Emotional Labor could be suggested.

The findings of this study regarding the effects of Emotional Expression are consistent with previous findings. The study showed moderating effects of the sub-dimensions of Emotional Intelligence on Emotional Labor and how they affect structural relationships among variables of Emotional Labor. Based on the findings of this study, two suggestions can be made.

Firstly, organizations in the service industry should provide training programs for their employees to improve their communication skills with the customers at the service contact points. The program should be divided into two categories: Verbal and Non-Verbal. As the importance of Non-Verbal communication was emphasized in the result, the training program to improve employees’ non-verbal communication skills would be more important. Further, it should be noted that communication within the organization would be as important as the communication between employees and customers.

Secondly, it would be important to actively include customers in the communication process at the service contact points. The characteristic of non-separability in service could be utilized to include customers in the communication process. Redesigning the service process and introducing various motivating incentives may enhance customer participation as well. By including customers more actively in the service process, organizations could build more positive and trusting emotions with customers.

The findings of this study would suggest the solutions that could enhance job satisfaction of the workers in the service industry with higher turnover rate, and in turn will increase the job security. The sustainability of workers’ lifestyle as well as the quality of their lives are, therefore, expected to be enhanced. Further, more effective management of the emotional labor would help organizations establish more positive organizational culture and enhance effectiveness of job performances, which will eventually increase the organizations’ profitability.

Lastly, this research shall provide meaningful results that confirm the importance of individual emotional intelligence. These results will help the organizations and individual workers understand how enhancing emotional intelligence will strengthen their adaptiveness to rapidly changing socioeconomic environments.

### Limitations and Future Directions

This study has a few limitations. Firstly, as this study focused on showing the moderating effects of the sub-dimensions of Emotional Intelligence, the data used in this study didn’t take industry-specific characteristics into account. Future studies may collect industry-specific data, such as banking or hospitality industry, in order to provide more in-depth findings of the moderating effects of emotional intelligence on how emotional expressions and perception of customer feedbacks affect employees’ Turnover Intention.

Further, the data used in this study was collected in the United States only and hence make it difficult for us either to generalize our findings or to conduct cross-country comparisons. Future studies may replicate the findings of this study with data collected from other countries or regions to enable cross country comparisons of the moderating effects of emotional intelligence.

Lastly, only the existence of the moderating effects of emotional intelligence was examined in this study. How employees’ emotional intelligence can be enhanced and thus help them manage emotional exhaustion should be more thoroughly studied, maybe in cultural contexts as well, in order to provide meaningful solutions to increase organizational effectiveness and sustainability. In order to do this, future studies may examine how emotional intelligence affects a person’s ability to empathize and to communicate.

## Conclusion

The result of this study provides the support for moderating effect of emotional intelligence on how employees’ Deep Acting and Surface Acting recognize customers’ reactions that would affect employees’ Emotional Exhaustion and Job Satisfaction, and hence, Turnover Intention. The result suggests that an employee with better understanding of his/her own emotions experience greater negative effect when there is a discrepancy between what they feel and how they should act.

Emotional Intelligence affects employees and the organizations in various ways. Although an employee with higher Emotional Intelligence experiences a greater effect on Job Satisfaction from Emotional Exhaustion, the one with a high Emotional Intelligence is more likely to recover from psychological frustration and emotional depletion and therefore would have lower Turnover Intention.

In addition, the ability to utilize his/her own emotions would enhance Job Performance of employees.

## Ethics Statement

An ethics approval was not required as per institutional guidelines and national laws and regulations because no unethical behaviors existed in this study. We just conducted paper–pencil test and were exempt from further ethics board approval since this research did not involve human clinical trials or animal experiments. All subjects gave written informed consent in accordance with the Declaration of Helsinki. Research respondents were ensured confidentiality and anonymity. All participation was voluntary.

## Author Contributions

YL and SL contributed to design models and hypotheses. YL and JC acquired, analyzed, and interpreted the data. YL and SL wrote and revised the article. YL, JC, and SL approved the final version.

## Conflict of Interest Statement

The authors declare that the research was conducted in the absence of any commercial or financial relationships that could be construed as a potential conflict of interest.
